# Detection of *KRAS, NRAS* and *BRAF* Mutations in Liquid Biopsy from Patients with Colorectal Cancer

**DOI:** 10.32604/or.2025.070116

**Published:** 2026-01-19

**Authors:** Katerina Ondraskova, Matous Cwik, Ondrej Horky, Jitka Berkovcova, Jitka Holcakova, Martin Bartosik, Tomas Kazda, Klara Mrazova, Michal Uher, Igor Kiss, Roman Hrstka

**Affiliations:** 1 Research Centre for Applied Molecular Oncology (RECAMO), Masaryk Memorial Cancer Institute, Zluty kopec 7, Brno, 656 53, Czech Republic; 2 National Centre for Biomolecular Research Faculty of Science, Masaryk University, Kamenice 5, Brno, 625 00, Czech Republic; 3 Department of Comprehensive Cancer Care and Faculty of Medicine, Masaryk Memorial Cancer Institute and Masaryk University, Zluty kopec 7, Brno, 656 53, Czech Republic; 4 Department of Pathology, Masaryk Memorial Cancer Institute, Zluty kopec 7, Brno, 656 53, Czech Republic; 5 Department of Radiation Oncology, Masaryk Memorial Cancer Institute, Zluty kopec 7, Brno, 656 53, Czech Republic; 6 Department of Biochemistry, Faculty of Science, Masaryk University, Kamenice 5, Brno, 625 00, Czech Republic; 7 Department of Health Information, Masaryk Memorial Cancer Institute, Zluty kopec 7, Brno, 656 53, Czech Republic

**Keywords:** Liquid biopsy, colorectal cancer (CRC), droplet digital PCR (ddPCR), *Kirsten Rat Sarcoma Viral Proto-Oncogene* (*KRAS*) mutation

## Abstract

**Objectives:**

Cancer treatment relies heavily on accurate diagnosis and effective monitoring of the disease. These processes often involve invasive procedures, such as colonoscopy, to detect malignant tissues, followed by molecular analyses to determine relevant biomarkers. This study aimed to evaluate the clinical performance of droplet digital PCR (ddPCR) for detecting *Kirsten Rat Sarcoma Viral Proto-Oncogene* (*KRAS*), *Neuroblastoma RAS Viral Oncogene Homolog* (*NRAS*), and *B-Raf Murine Sarcoma Viral Oncogene Homolog B* (*BRAF*) mutations in circulating tumor DNA (ctDNA) from colorectal cancer patients using liquid biopsy.

**Methods:**

ctDNA was isolated from colorectal cancer (CRC) patients (n = 110) and analyzed for *KRAS*, *BRAF*, and *NRAS* mutations. The ctDNA obtained through liquid biopsy was analyzed using ddPCR, and the findings were compared with sequencing data from tumor DNA archived in formalin-fixed paraffin-embedded (FFPE) blocks.

**Results:**

For *KRAS* mutations, ddPCR achieved a sensitivity of 72.0% and a specificity of 71.4%. However, when pooling all target mutations (*KRAS, NRAS* and *BRAF*), the overall sensitivity and specificity were lower, at 48.3% and 51.1%, respectively.

**Conclusion:**

The results of this study indicate that the ddPCR analysis of ctDNA may provide complementary information for the molecular diagnosis of CRC patients.

## 1 Introduction

Accurate diagnosis is the basis of cancer treatment, beginning with the identification of malignant tissue present in a patient [1]. The usual way to confirm the presence and characteristics of cancer is through tissue biopsy analysis, where tumor cells exhibit distinct properties from healthy cells. These differences arise due to genetic changes conferring a selective advantage and accumulating throughout tumor progression. These mutations may serve as important predictive and prognostic factors in oncological practice [2]. However, traditional tissue biopsy procedures are invasive and may capture only a limited and non-representative fraction of tumor cells. Moreover, excised tumor tissues do not reflect the dynamic nature of the tumor microenvironment and may differ significantly from residual tumor tissue, which can serve as a source of recurrence. This has led to research on other potential ways for cancer detection—one of them being analysis of liquid biopsies (LBs) [3].

Liquid biopsy offers a non-invasive technique to analyze body fluids (most often blood or urine), including components such as various protein biomarkers, circulating cell-free DNA (cfDNA), circulating tumor DNA (ctDNA), circulating tumor cells (CTCs) and many others [4]. LBs allow monitoring of patients with cancer, especially based on DNA or RNA analysis, and represent an alternative when tissue biopsies cannot be obtained or are complicated, such as colonoscopy. Circulating tumor DNA, a fraction of cell-free DNA originating specifically from tumor cells, provides an innovative approach for cancer monitoring [5]. Specifically, these small DNA fragments with tumor-specific mutations are released into the blood and constitute only a minor part of the total cfDNA. According to recent studies, ctDNA accounts for less than 1% of an individual’s total cfDNA [6,7]. Previous advancements in assay sensitivity and refinement have established ctDNA analysis as a reliable tool in the field of oncology [8,9]. Therefore, ctDNA can be used as a prognostic and diagnostic biomarker due to the correlation between the stage of cancer and other factors, such as location, tumor progression, and pattern of metastasis [10,11]. In the last decade, there has been a significant improvement in the analysis of cfDNA and, consequently, ctDNA. Next-generation sequencing (NGS) and digital PCR (dPCR) are some of the most frequently used methods for detecting cfDNA and ctDNA in liquid biopsy, mainly due to the high specificity and sensitivity [12]. Among these, droplet digital PCR (ddPCR) offers additional advantages, such as absolute quantification, high precision, and the ability to detect low-frequency mutations, making it especially valuable for monitoring minimal residual disease and treatment response.

Colorectal cancer (CRC) is one of the most frequently diagnosed malignancies worldwide and currently ranks third in terms of incidence. In addition, CRC is the second leading cause of cancer-related mortality worldwide. In 2022, according to the GLOBOCAN database, more than 1.9 million cases of CRC were recorded. There is a stable rise in the incidence of CRC among adults younger than 50 years, and it has been rising in developing countries as well [13]. A substantial number of patients (approximately 25%) are still diagnosed with metastatic disease and an additional 25%–50% of patients with early-stage disease will develop metastases later [14–17]. The treatment and prognosis of metastatic CRC remain difficult, with a 5-year relative survival rate of only ~16% in the US [18]. Thus, there is a need to improve screening, diagnostics, and treatment. Standard screening and/or diagnostic tools include fecal occult blood tests (FOBTs), colonoscopy or CT scan.

Current metastatic CRC treatment involves biomarker testing, including *RAS* mutations [19], *BRAF*^V600E^ mutations and microsatellite instability (MSI) status [20]. The presence of *RAS* mutations predicts a lack of response to anti-EGFR treatments, while *BRAF*^V600E^ mutation is a negative prognostic marker. However, it is a positive predictive marker for novel *BRAF*^V600E^—targeted therapies [21]. MSI status has prognostic and predictive value for responsiveness to modern immunotherapy with checkpoint inhibitors. *Kirsten Rat Sarcoma Viral Proto-Oncogene* (*KRAS*) is a gene located on chromosome 12 that provides signals allowing the formation of the K-Ras protein. Activated *KRAS* mutations drive cell proliferation, suppress differentiation and are crucial for the initiation of carcinogenesis. K-Ras, a membrane-anchored GTP/GDP-binding protein, is responsible for the transmission of signals from the cytosol to the cell nucleus. It is also part of the important MAPK/ERK signaling pathway [19,20,22,23]. *Neuroblastoma RAS Viral Oncogene Homolog* (*NRAS*) mutations are associated with tumor development and the protection of cells from apoptosis [24]. They occur in only 3%–5% of all CRCs, making them less frequent than *KRAS* or *BRAF* mutations, with which they are also believed to be incompatible [25]. However, the importance of *NRAS* in CRC has increased owing to its possible role in the clinical management of advanced stages of this disease [26].

The effect of CRC treatment seems to be closely related to the mutation status of the *B-Raf Murine Sarcoma Viral Oncogene Homolog B* (*BRAF*) gene and MSI status. CRC patients with MSI-stable carcinoma and the presence of a mutation in the *BRAF* gene show shorter overall survival (OS) than patients without the mutated gene [27,28]. In addition to *KRAS* mutations, *BRAF* mutations are also recognized as critical factors influencing the efficacy of anti-EGFR targeted therapy, being responsible for worse prognosis and reduced response to chemotherapeutic agents [29].

This retrospective study evaluates the diagnostic potential of cfDNA obtained from liquid biopsy using ddPCR in the molecular diagnosis of CRC to determine the benefit of ctDNA-based diagnosis compared to standard tissue biopsy. We determined the sensitivity, specificity, and overall efficiency of ddPCR for the detection of the most common *KRAS* gene point mutations at the major hotspot codons G12 and G13. We also identified mutations in *BRAF* and *NRAS*, which are frequently present in CRC, especially in cases with wt *KRAS*. We optimized the method using selected cancer cell lines containing mutations in these genes (more information is provided in the Methods section). Following this, we tested samples from patients with CRC to explore its applicability in molecular diagnostics, monitoring of minimal residual disease, therapeutic decision-making, and ultimately the quality of life of patients in the future.

## 2 Materials and Methods

### 2.1 Cell Lines

The cell lines (Caco-2, HCT116, PANC-1, DLD-1, MIA PaCa-2, SW480, HT-29 and MOLT-4) used to optimize the detection of selected mutations by ddPCR (Table A1) were obtained from the American Type Culture Collection (ATCC). They were maintained in medium as indicated (all media were from Merck & Co., Inc., Rahway, NJ, USA), supplemented with 10% fetal bovine serum (Life Technologies Corp., Carlsbad, CA, USA), 1% pyruvate and L-glutamine at 37°C in a humidified atmosphere of 5% CO_2_. Subcultures were prepared when cells reached 75% confluence, roughly every 2–3 days. All cell lines were regularly tested for mycoplasma contamination with consistently negative results, and those obtained more than two years ago additionally underwent authentication by short tandem repeat (STR) profiling. These cell lines served as positive controls to validate the ddPCR assays and to ensure assay specificity and accuracy.

### 2.2 Genomic DNA Extraction and Fragmentation

Genomic DNA (gDNA) was isolated from individual cell lines using the DNeasy Blood & Tissue Kit (Cat. No. 69504 QIAGEN N.V., Venlo, The Netherlands). The concentration of the eluted gDNA was measured using a Qubit™ 4 Fluorometer (Thermo Fisher Scientific, Waltham, MA, USA) and Qubit™ dsDNA BR Assay Kit (Cat. No. Q33265 Thermo Fisher Scientific, Waltham, MA, USA). gDNA was then diluted to achieve a concentration of 2 µg/µL and subjected to fragmentation using a high-capacity ultrasonicator, Covaris M220 (Covaris, LLC, Woburn, MA, USA). The process was recorded using a SonoLab7 system (Covaris, LLC, Woburn, MA, USA), with Snap-Cap micro-TUBE tubes (Sigma-Aldrich, Burlington, MA, USA). There were 200 cycles during the fragmentation of each sample.

### 2.3 Liquid Biopsies

Venous blood samples from CRC patients treated at Masaryk Memorial Cancer Institute (Table 1) were collected between 2011 and 2020 in Sarstedt tubes containing a clotting activator, allowed to clot at room temperature for 45 min, and subsequently centrifuged to obtain serum. The extracted serum was immediately stored at −20°C and moved to −80°C within two weeks. The samples were then stored in the Bank of Biological Material at Masaryk Memorial Cancer Institute (BBM MMCI) at −80°C. Out of the total 110 patients, 39 were women with a median age of 64.5 years and 71 were men with a median age of 66.0 years. An additional cohort of 30 control samples (40% female, 60% male) was collected from volunteers who underwent preventive cancer screening at MMCI to test the specificity of ddPCR. All participants enrolled in the study signed an informed consent to provide samples for research purposes and the study was approved by Ethics Committee of the Masaryk Memorial Cancer Institute (2020/2489/MOU). To minimize degradation and freeze–thaw artifacts, all serum aliquots were subjected to a single freeze-thaw cycle prior to cfDNA extraction. Hemolytic samples were excluded based on visual inspection. cfDNA was isolated using the Plasma/Serum Cell-Free Circulating DNA Purification Mini Kit (Cat. No. SKU 55100 Norgen Biotek Corp., Thorold, ON, Canada) and its concentration was measured using the Qubit™ dsDNA HS Assay Kit (Cat. No. Q33230 Thermo Fisher Scientific, Waltham, MA, USA), which also served as a basic cfDNA integrity metric. The concentrations of isolated cfDNA ranged from 0.08 to 33.90 ng/µL. All pre-analytical processes were carried out in accordance with the general guidelines for the handling of cell-free nucleic acids, including the ISO 20186-3:2019 recommendations. Recent evidence emphasizes the critical importance of standardized pre-analytical workflows in minimizing cfDNA degradation and pre-analytical variability, particularly regarding serum vs. plasma handling, centrifugation timing, and temperature control [30].

**Table 1:** Patient medical data

Basic characteristics of patients and primary tumor (n = 110 patients)		Descriptive statistics*
**Sex**	Female	39 (35.5%)
	Male	71 (64.5%)
**Age at surgery**	[years]	64.5 ± 10.7 66.0 (58.2; 73.0)
**Localization of the primary tumor**	cecum (C18.0, C18.1)	20 (18.2%)
	colon (C18.2–C18.7, C18.9)	28 (25.5%)
	lesions extending beyond the colon (C18.8)	8 (7.3%)
	rectosigmoideal connection (C19)	22 (20.0%)
	rectum (C20)	32 (29.1%)
**Extent of tumor (T)**	T1	1 (0.9%)
	T2	8 (7.3%)
	T3	72 (65.5%)
	T4	27 (24.5%)
	Tx	2 (1.8%)
**Metastases in the lymph nodes (N)**	N0	37 (33.6%)
	N1	34 (30.9%)
	N2	37 (33.6%)
	Nx	2 (1.8%)
**Distant metastases (M)**	M0	57 (51.8%)
	M1	47 (42.7%)
	Mx	6 (5.5%)
**Lymphovascular space invasion (L)**	L0	62 (56.4%)
	L1	35 (31.8%)
	Lx	13 (11.8%)
**Grade**	G1	27 (24.5%)
	G2	60 (54.5%)
	G3	18 (16.4%)
	G4	1 (0.9%)
	Gx	4 (3.6%)
**Clinical stage**	stage I	3 (2.7%)
	stage II	24 (21.8%)
	stage III	36 (32.7%)
	stage IV	47 (42.7%)
**Neoadjuvant therapy**	no	93 (84.5%)
	yes	17 (15.5%)

Note: *Mean ± standard deviation and median (interquartile range) are given for continuous variables (age) and absolute (relative) frequencies are given for categorical variables.

### 2.4 NGS Analysis

The percentage of neoplastic cells in formalin-fixed paraffin-embedded (FFPE) tissues was estimated by a pathologist, and the minimum content of tumor cells in the selected area required for molecular analysis was 10%. DNA was extracted using the cobas® DNA Sample Preparation Kit (Cat. No. 05985536190, Roche, Indianapolis, IN, USA) or the QIAamp DNA FFPE Tissue Kit (Cat. No. 56404 QIAGEN N.V., Venlo, The Netherlands).

Mutational analysis was performed by NGS using commercially available kits according to manufacturer’s protocols: EliGene Colorectum NGS (Cat. No. 90066-NGS Elisabeth Pharmacon, Brno, Czech Republic) or Accel-Amplicon Plus Colorectal Cancer Panel (Cat. No. AP-CR8048, Swift Biosciences, Ann Arbor, MI, USA) as an amplicon sequencing approach, or hybridization-based Sequence Capture targeted enrichment of custom-defined regions (Roche NimbleGen, Inc., Pleasanton, CA, USA). Sequencing was performed using a MiSeq instrument (Suite 4.1.0.480, Illumina, San Diego, CA, USA). The mutation status was assessed using NextGENe software 2.4.2.2 (Softgenetics LLC, State College, PA, USA). The minimum allele frequency was set to 5% and the minimum coverage of sequenced regions (300×) was checked in Integrative Genomics Viewer 2.11.2 (IGV 2.11.2, Broad Institute, Cambridge, MA, USA) and/or NextGENe. All three NGS panels covered the hotspot regions subsequently assessed by ddPCR (*KRAS* codons 12/13, *NRAS* codon 61, and *BRAF* V600E). No systematic differences in mutation detection were observed between the sequencing platforms, and NGS results across platforms were deemed comparable reference standards.

### 2.5 Droplet Digital PCR (ddPCR)

**Figure 1:**
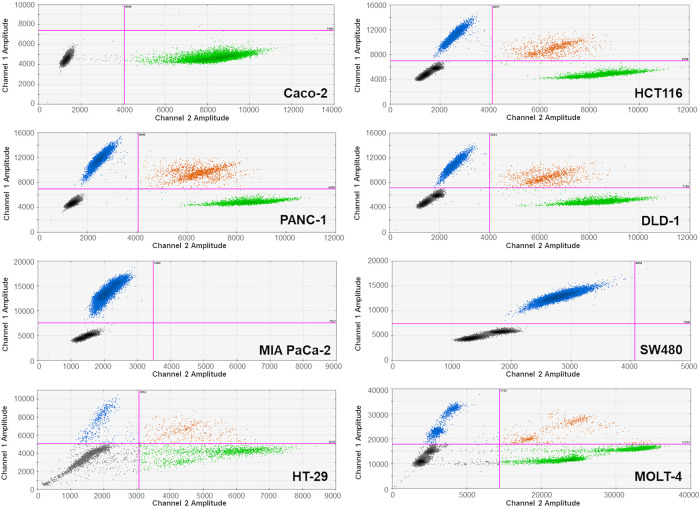
Representative ddPCR graphs of genomic DNA (gDNA) from tested cancer cell lines. Droplet populations are color-coded as follows: blue—droplets containing mutant alleles; orange—droplets containing both mutant and wild-type alleles; green—droplets containing only wild-type alleles; black—negative droplets (no target DNA). Droplet clusters were manually gated using threshold lines (purple). Mutation-specific assays were applied as follows: KRAS hotspot mutations (codons G12/G13) were analyzed in all cell lines except HT-29 and MOLT-4; BRAF V600E mutation was analyzed in the HT-29 cell line; and NRAS G12C mutation in the MOLT-4 cell line

We focused on detecting the most frequent and clinically relevant hotspot mutations in *KRAS* (codons 12 and 13), *NRAS* (codon 61), and *BRAF* (V600E), as they represent the majority of actionable mutations associated with anti-EGFR therapy resistance in colorectal cancer. Other *KRAS* codons and non-V600E *BRAF* mutations, although biologically relevant, were not included due to their low prevalence (<5%) and limited impact on treatment algorithms as supported by [28,29]. For the analysis, we used commercial kits produced by Bio-Rad Laboratories, Inc. (Hercules, CA, USA): the ddPCR™ KRAS G12/G13 Screening Kit (1863506), ddPCR™ BRAF V600 Screening Kit (12001037), and ddPCR™ NRAS Q61 Screening Kit (12001006). The method was optimized and validated using cancer-derived cell lines differing in *KRAS*, *BRAF* or *NRAS* gene mutation statuses (Table A1), which served as positive and negative controls for each assay. These reference cell lines were used to define the droplet amplitude thresholds and confirm the accuracy of the mutation detection. Droplet clustering patterns were confirmed in each run using these controls, as illustrated in Fig. 1.

The reaction mix contained 11 µL of 2× ddPCR Supermix for Probes (No dUTP), 1 µL of 20× Multiplex Primer FAM + HEX (all Bio-Rad Laboratories, Inc., Hercules, CA, USA), and 4.8–5 µL of deionized water depending on whether 0.2 µL of restriction enzyme *MseI* (*KRAS* mutation, New England Biolabs, Ipswich, MA, USA) or *HindIII* (*NRAS* and *BRAF* mutations, MBI Fermentas, Amherst, NY, USA) was added to the mix in the case of genomic DNA. The mixture was then pipetted into a High-Profile 96-Well PCR plate (Bio-Rad Laboratories, Inc., Hercules, CA, USA) and 5 µL of cfDNA was added to a final volume of 22 µL per well. Subsequently, the plate was sealed by PX1™ PCR Plate Sealer (Bio-Rad Laboratories, Inc., Hercules, CA, USA) and inserted into an Automated Droplet Generator system (SN 773BR1017, Bio-Rad Laboratories, Inc., Hercules, CA, USA) to prepare a water-oil emulsion of the samples. PCR was performed in the C1000 Touch™ Thermal Cycler (Bio-Rad Laboratories, Inc., Hercules, CA, USA) according to the manufacturer’s protocol (Table A2). The plate was then inserted into the QX200 Droplet Reader (Bio-Rad Laboratories, Inc., Hercules, CA, USA) device to analyze all droplets using a two-color detection system. The results were plotted on a graph of fluorescence intensity using QuantaSoft™ Software Version 1.7.4 (Bio-Rad Laboratories, Inc., Hercules, CA, USA). Droplet amplitude thresholds were manually defined with reference to the positive and negative controls for each run (Fig. 1). The threshold line was set above the highest cluster of negative droplets and below the lowest cluster of positive droplets, in accordance with the Bio-Rad guidelines.

To ensure that the performance of the measurement procedure is consistent with detection capability, we determined both the Limit of Blank (LoB) and the Limit of Detection (LoD) for the ddPCR assay. The LoB was established by performing 20 measurements using gDNA (wt *KRAS* gene), with 18 out of 20 results falling below the LoB threshold of 0.4 copies/µL. This corresponds to a 90.0% proportion with an associated upper one-sided 95% confidence interval of 96.6%.

To define the sensitivity of detection, we conducted serial dilutions of genomic DNA derived from the DLD-1 cell line (*KRAS* G13D mutation), as summarized in Table A3. Three independent dilution experiments were performed in three technical replicates each. The minimal detectable input of cfDNA in the ddPCR reaction was determined to be approximately 1.76 ng/µL. A minimum of two positive droplets (i.e., ≥0.4 copies/µL) was required to classify a sample as mutation-positive. These empirically derived thresholds were used to interpret patient samples and ensured that only signals above the assay-specific detection limit were considered true positives.

To assess inter-operator reproducibility, two independent analysts reviewed 110 ddPCR results. Operator 1 identified 67 samples as mutant and 43 as wild-type. Operator 2 classified 66 of the 67 mutant samples identically and all 43 wild-type samples as such, with only one discordant call. Cohen’s κ statistic was 0.981, confirming excellent agreement and high robustness of the classification process. A cell line displaying the tested mutation (Table A1, Fig. 1) was used as a positive control for each cfDNA analysis. Deionized water was used as a negative control.

### 2.6 Statistical Analysis

Standard descriptive statistics were used to describe the analyzed cohort: mean, standard deviation, median and interquartile range are given for continuous variables and absolute and relative frequencies are given for categorical variables. The diagnostic accuracy of mutation detection by ddPCR was evaluated using sensitivity, specificity, predictive values and, using receiver operator characteristic (ROC) analysis, the overall accuracy was summarized and tested as the area under the curve (AUC). A univariate logistic regression model was used for the basic assessment of the association of clinical and pathological features with mutational status, while a multivariate model was used to estimate odds ratios (OR) adjusted for other variables. Statistical significance testing of the AUC and ORs was performed at the 5% significance level. All calculations were performed in IBM SPSS Statistics software version 25 (IBM, Armonk, NY, USA).

## 3 Results

### 3.1 Optimization of ddPCR

To assess the sensitivity of ddPCR (i.e., to what extent the *KRAS* mutation can still be detected by this method), dilution series of genomic DNA (gDNA) were prepared. The limit of detection (LoD) was determined using isolated gDNA from the DLD-1 (heterozygous KRAS G13D) cell line. Initial input amount was 400 ng/µL of DNA (100%, final concentration of 11.429 ng/µL), and subsequent dilutions were prepared to final concentrations of 25%, 6.25%, 1.56%, 0.39%, 0.098% and 0.024% (Table A3). ddPCR analysis confirmed that positive and negative droplets could still be clearly distinguished even at low gDNA input levels, demonstrating high assay sensitivity. Based on the dilution series, our results indicate that the minimal reliably detectable concentration is approximately 0.04 ng/µL. Subsequently, we analyzed a panel of cell lines with known mutation status (mutant, wild-type, or heterozygous; Table A1) in order to verify the assay’s selectivity and to define the amplitude ranges for both wild-type and mutant droplets. Considering these findings and the determined Limit of Blank, a threshold value of ≥0.04 copies/µL was established to classify samples as positive for mutation detection.

### 3.2 Samples from Patients with CRC

A total of 110 patients with primary colorectal cancer were included in this study, of whom 47 patients (42.7%) had metastatic disease. Patients were treated according to the standard of care. All relevant clinical, demographic, and epidemiological data were collected from each patient (Table 1). As a control group, we used healthy patients participating in a cancer prevention program at the Masaryk Memorial Cancer Institute (30 patients) who did not have cancer or any severe health problems. Mutational status of *KRAS/BRAF/NRAS* genes in the primary tumor was determined by NGS, applying either an amplicon or hybridization-based strategy, and the results were compared with the mutational status identified using ctDNA analysis by ddPCR from the peripheral blood serum of the respective patients (Table 2). No systematic differences in mutation detection were observed between the platforms, and all provided concordant results with the ddPCR assays. Briefly, 63 tumor samples were mutated (57.3%) in at least one of the analyzed genes, and 47 samples were wild-type (42.7%) using the standard of care predictive pathology tissue examination via NGS. For comparison, analysis of ctDNA from liquid biopsies revealed that 68 samples were mutated (61.8%) and 42 samples were wild-type (38.2%). The most abundant mutation in both cases was the *KRAS* gene mutation, which was present in 50 samples by NGS and 67 samples by ddPCR (45.5%; 60.9%). Other mutations were present in the following order: *BRAF*^V600E^ mutation was present in 9 and 5 samples, respectively (8.2%; 4.5%), and *NRAS* mutation was found in 4 and 3 samples, respectively (3.6%; 2.7%). One sample showed concomitant mutations detected by ctDNA analysis. All liquid biopsy samples were primarily tested for the presence of the *KRAS* mutation. The majority of samples with confirmed *KRAS* mutation were not further tested for the presence of *BRAF* and *NRAS* mutations (Table A4), as the primary aim of the study was to determine any mutation and use it for subsequent disease monitoring.

**Table 2:** Determination of *KRAS*, *BRAF* and *NRAS* mutations

Gene		NGS	ddPCR
*KRAS*	mut	50 (45.5%)	67 (60.9%)
	wt	60 (54.5%)	43 (39.1%)
	NA	0 (0.0%)	0 (0.0%)
*BRAF*	mut	9 (8.2%)	5 (4.5%)
	wt	99 (90.0%)	28 (25.5%)
	NA	2 (0.9%)	77 (70.0%)
*NRAS*	mut	4 (3.6%)	3 (2.7%)
	wt	105 (95.5%)	10 (9.1%)
	NA	1 (0.9%)	97 (88.2%)
*KRAS*/*BRAF*/*NRAS*	mut	63 (57.3%)	68 (61.8%)
	wt	47 (42.7%)	42 (38.2%)
	NA	0 (0.0%)	0 (0.0%)

Note: The absolute (relative) frequencies of the test results are provided, including the unperformed tests (especially for the ddPCR method); NGS, next generation sequencing; ddPCR, droplet digital PCR; mut, mutation; wt, wild-type; NA, not available.

Overall, the mutation status determined in tumor tissue and in ctDNA did not show complete concordance, as illustrated by several discordant cases (Table 2). Conversely, there were also patients in whom mutations were present in the tumor tissue but not detectable in ctDNA. Importantly, no mutations were detected in the control group. Previous studies have also demonstrated that disagreement between tissue and plasma-based detection of KRAS mutations may occur and may influence treatment decisions. For example, the IRON group reported an 85% concordance (29/34 patients) using chip-based digital PCR, with sensitivity and specificity of 69% and 100%, respectively [31]. Similarly, the PREDATOR study detected KRAS mutations in plasma not always observed in tissue, likely reflecting tumor heterogeneity or clonal evolution [32]. Several other studies confirm discordance rates of 10%–20% in mCRC cohorts, often due to low ctDNA burden or sampling bias [33,34]. These findings highlight the need for orthogonal validation, such as ddPCR for hotspot regions, which we employed to ensure consistency across NGS platforms.

A detailed comparison of concordant and discordant cases is provided in Tables A5 and A6, which summarize the tumor stage, neoadjuvant treatment status, and tissue–blood sampling interval. Discordant results occurred more frequently among patients who received neoadjuvant therapy and those with longer intervals between blood and tissue sampling. The ddPCR method achieved a statistically significant but only modest discrimination performance when detecting KRAS mutations, with an AUC = 0.602 (*p* < 0.026), sensitivity of 72.0%, and specificity of 48.3% (Table 3). When all three target mutations were included in the analysis, the overall accuracy improved slightly (AUC = 0.612; *p* = 0.016) accompanied by comparable sensitivity (71.4%) and a minor increase in specificity (51.1%) (Table 3). These results indicate that while ddPCR can detect a substantial fraction of mutation-positive cases, its ability to correctly exclude mutation-negative patients is limited. The subgroup analyses stratified by clinical stage (I–IV) and mutation class are summarized in Table A7, showing improved sensitivity in stage IV disease but persistently low specificity across all subgroups. Briefly, in early-stage disease (stage I–II), the diagnostic performance was modest, with a sensitivity of 66.7% and a specificity of 55.6%. In stage III, the accuracy remained limited, with sensitivities of approximately 55.6%–58.3% and specificities of 50.0%–58.3%, depending on whether only *KRAS* or any *KRAS*/*BRAF*/*NRAS* mutation was considered. In contrast, the performance improved in advanced disease: in stage IV, the sensitivity increased to 87.0% for *KRAS* mutations and 83.3% for *KRAS*/*BRAF*/*NRAS*, albeit at the cost of reduced specificity (41.7% and 41.2%). In the subgroup of patients without neoadjuvant therapy (n = 93), the sensitivity reached 78.6% for *KRAS* mutations, with a specificity of 52.9%.

**Table 3:** Evaluation of the diagnostic accuracy of *KRAS* or any *KRAS/BRAF/NRAS* mutation detection by ddPCR

Metric	KRAS mutation	KRAS/BRAF/NRAS mutation
Area under the curve (AUC)	0.602 (0.691; 0.512)	0.612 (0.521; 0.704)
AUC with bootstrapped CI	0.602 (0.513; 0.690)	0.612 (0.522; 0.703)
Sensitivity	72.0 (58.1; 82.7)%	71.4 (59.1; 81.2)%
Specificity	48.3 (36.1; 60.8)%	51.1 (37.1; 64.9)%
Positive predictive value	53.7 (41.8; 65.2)%	66.2 (54.2; 76.4)%
Negative predictive value	67.4 (52.3; 79.7)%	57.1 (42.0; 71.1)%

Note: For all values, 95% CI is given; ddPCR, droplet digital PCR; CI, confidence interval.

### 3.3 Association of Clinical and Pathological Features with the Mutational Status

We evaluated which clinicopathological factors were significantly associated with the presence of the *KRAS* gene mutation (Table A8). *KRAS* mutations were identified with greater frequency in rectal (36.0% vs. 23.3%) and cecal tumors (22.0% vs. 15.0%), whereas tumors at the rectosigmoid junction were more prevalent in the *wild-type* group (28.3% vs. 10.0%). However, these differences were not statistically significant. Other relevant findings included a higher proportion of N2 lymph node metastases among patients in the *KRAS-mutated* group (42.0% vs. 26.7%) and a trend towards a higher prevalence of clinical stage IV cases (46.0% vs. 40%). Although not statistically significant, these observations may suggest increased tumor aggressiveness associated with the presence of the *KRAS* mutation [35].

When extending the analysis to include any of the *KRAS*/*BRAF*/*NRAS* mutations (Table A9), the association with cecal tumor localization became statistically significant (25.4% vs. 8.5%, *p* = 0.009). Moreover, patients harboring any of these mutations were more often diagnosed at clinical stage IV (47.6% vs. 36.2%, *p* = 0.017) and again showed a trend towards a higher prevalence of N2 lymph node metastases (41.3% vs. 23.4%, *p* = 0.049). Taken together, the presence of *KRAS/BRAF/NRAS* mutations was associated with more aggressive disease features, particularly in cecal tumors, consistent with recent evidence linking mutation status to tumor localization and nodal involvement [36].

## 4 Discussion

Circulating nucleic acids represent a promising new type of biomarker with the potential to improve cancer treatment in many aspects in the future. One of the aims of this study was to evaluate the use of ddPCR in the detection of somatic mutations present in the tumor tissue of CRC patients. Tissue-based mutation analysis is limited by certain characteristics of cancer, particularly the spatial and temporal heterogeneity of tumor tissue [35]. Moreover, for patients where tumor tissue sample is difficult or impossible to obtain, ctDNA-based testing might provide a non-invasive solution for the purpose of initial molecular diagnosis. The percentage of CRC patients in whom ctDNA can be detected depends on the extent of the disease and ranges from 50% in patients with non-metastatic disease to nearly 90% in patients with metastatic disease [37]. Therefore, ctDNA analysis could have applications in screening, molecular diagnosis of cancer, risk stratification of disease relapse after initial curative intent treatment or monitoring of cancer treatment success in a palliative setting [38]. Evidence from multiple trials indicates a high consistency (>90%) between ctDNA and standard tumor tissue-based *RAS* testing [11].

Among the available technologies for ctDNA analysis, ddPCR has emerged a sensitive and specific method for detecting tumor-associated mutations [38]. However, its use in clinical practice remains limited [39]. This makes liquid biopsy and ddPCR-based ctDNA analysis an increasingly attractive complementary approach, particularly as a faster alternative for patients requiring urgent treatment decisions or those whose molecular profile restricts therapeutic options. Beyond its non-invasive nature, liquid biopsy also enables longitudinal monitoring of tumor evolution and better captures the heterogeneity of primary tumors and metastatic lesions [35]. Building on this rationale, our results demonstrate that ddPCR is a robust and sensitive method for detecting *KRAS* mutations in ctDNA, although its specificity was moderate. The slight improvement in overall accuracy when combining multiple mutations suggests that expanding the mutation panel may help to partially compensate for the limited specificity inherent to ctDNA analysis in early-stage disease or low-tumor-burden settings. Although the diagnostic gain was incremental, these findings reinforce the utility of ddPCR not only in mutation screening but also in tracking minimal residual disease over time, where even small changes in ctDNA levels may be clinically relevant.

Despite achieving statistically significant discrimination, the diagnostic accuracy of ddPCR in our study was modest, with AUC values close to 0.6 and specificity consistently below 52%. These findings underscore important limitations of ddPCR as a stand-alone diagnostic tool in this setting. Several technical and biological factors likely contributed to the observed performance. First, serum rather than plasma was used for cfDNA isolation, and serum-derived samples are known to suffer from leukocyte DNA contamination that reduces assay specificity [40]. Second, a substantial proportion of patients had early-stage disease, where ctDNA shedding is often insufficient for reliable detection, as also shown by Bettegowda et al., who detected ctDNA in only 55% of localized cases [8]. Low ctDNA levels, together with limited assay sensitivity, likely explain cases where KRAS mutations were detected in tissue but not in ctDNA. Conversely, instances where mutations were found in cfDNA but not in tumor tissue may reflect tumor heterogeneity or spatial sampling limitations [41]. Biological variability introduced by neoadjuvant therapy and longer blood–tissue sampling intervals further contributed to discordant results, as effective treatment can reduce tumor burden and ctDNA levels to below detection thresholds [42,43]. Our subgroup analyses (Tables A5 and A6) support this interpretation, showing that discordant findings were more frequent in patients who had undergone neoadjuvant therapy and in those with longer sampling intervals.

In addition, certain methodological aspects may have influenced the results. The mutation panel was restricted to the hotspots covered by the selected Bio-Rad kits, precluding analysis of less frequent KRAS or BRAF variants. In addition, not all patients underwent ddPCR testing for *BRAF* and *NRAS* mutations. This was an intentional choice, as once a *KRAS* mutation was identified, further *NRAS*/*BRAF* testing was not performed in line with current diagnostic algorithms that consider these mutations largely mutually exclusive. While rare instances of concurrent mutations have been reported [44], their frequency remains low, and universal multi-gene testing in *KRAS-*positive cases is not yet routine.

Finally, clonal hematopoiesis of indeterminate potential may represent another important confounder. Somatic variants arising in hematopoietic stem cells can be released into circulation and misclassified as tumor-derived alterations [45,46]. Somatic mutations in hematopoietic stem cells most frequently involve genes such as *DNMT3A*, *TET2*, and *ASXL1*, but occasionally also affect genes involved in RAS/MAPK pathways. These mutations can be released into circulation and detected in cfDNA, potentially mimicking tumor-derived alterations [47]. Since our study did not include matched sequencing of leukocyte DNA, we cannot exclude the possibility that some of the mutations detected in serum samples originated from clonal hematopoiesis. Previous studies have shown that clonal hematopoiesis is common in older cancer patients and can confound liquid biopsy analyses if not accounted for [45,46].

It is noteworthy that our results are consistent with previously published data. For example, in the meta-analysis by Peng et al., the sensitivity of digital PCR methods to detect *KRAS* mutation in plasma samples of CRC patients ranged from 67% to 96% in 12 studies; however, the results varied greatly due to limited sample sizes and utilization of different digital PCR methods [48]. In another meta-analysis comprising 17 studies that used ddPCR to detect *KRAS* mutation in cfDNA, sensitivity ranged from 36% to 100% and specificity ranged from 50% to 100% [49]. Bettegowda et al. reported that the sensitivity of ctDNA for detection of clinically relevant *KRAS* gene mutations was 87.2% and its specificity was 99.2% using digital PCR in 206 metastatic colorectal cancer patients [8]. The higher sensitivity in the study by Bettegowda et al. can be explained by the fact that only patients with metastatic disease were included. Accordingly, we attempted to analyze only cases of metastatic CRC and the sensitivity increased to 87.0%. Likewise, a study focused on ctDNA analysis in patients divided according to tumor stage confirmed that ctDNA concentrations increase with tumor size and cancer stage. For example, CRC patients in stage I showed 47% sensitivity of *KRAS* mutation, in contrast to 87% sensitivity observed in patients in stage IV [50]. This assumption is also supported by a study by Liebs et al., who measured the mutations in matching tumor and plasma samples and observed the highest accuracy (68%) in patients with distant metastases, demonstrating that cfDNA analysis by ddPCR in patients with earlier stages of cancer is limited [51].

Interestingly, we observed that the presence of any *KRAS, BRAF*, or *NRAS* mutation was more prevalent in right-sided colorectal cancers, particularly those originating in the cecum (25.4% vs. 8.5%, *p* = 0.009). Moreover, patients with these mutations had a higher frequency of advanced nodal involvement (N2: 41.3% vs. 23.4%, *p* = 0.049) and a greater proportion of stage IV disease (47.6% vs. 36.2%, *p* = 0.017), suggesting an association between mutational status and more aggressive tumor behavior. These findings are in agreement with previous studies demonstrating that *RAS* and *BRAF* mutations are more common in right-sided colorectal cancers and are associated with poorer prognosis [21,52–55]. While our cohort was relatively small, the observed trends reinforce the clinical value of liquid biopsy in capturing actionable mutational patterns and guiding treatment decisions based on tumor location and molecular profile.

This study has several limitations that should be highlighted. (i) The relatively moderate specificity of ddPCR, particularly in early-stage CRC, which may be influenced by low ctDNA shedding and potential background noise in the assay. (ii) Not all ctDNA samples were tested for all three target genes (*KRAS, NRAS, BRAF*), which may limit the overall diagnostic yield and assessment of combinatorial mutation profiles. (iii) Discrepancies between ctDNA and tissue-based NGS results could reflect tumor heterogeneity, sampling bias, and/or the effects of neoadjuvant therapy. (iv) The study was conducted at a single institution, and the relatively small cohort size may restrict the generalizability of the findings to broader patient populations.

## 5 Conclusions

Liquid biopsy collection followed by ctDNA analysis represents a promising alternative to the standard care examination of tissue obtained by biopsy. Its use is particularly suggested for monitoring cancer relapse after primary treatment or as a tool to assess the response to treatment in patients with metastasis by monitoring minimal residual disease through the detection of specific tumor mutations in peripheral blood.

Our data support the potential of ddPCR being a reliable and sensitive method for the detection and analysis of ctDNA in molecular diagnosis of colorectal cancer. However, the routine use of this approach in the management of CRC patients remains to be widely applied in clinical practice. Herein lies the importance of our work, which demonstrates that liquid biopsy may represent a valuable complement to standard tissue-based biopsy in molecular diagnosis of CRC patients because it overcomes the problem of tumor heterogeneity and allows detection of mutations that may be missed due to spatial and temporal subsampling of tumor tissue or due to a minor number of mutated cells. Thus, we conclude that liquid biopsy and ctDNA analysis by ddPCR may represent a complementary tool to standard tissue-based biopsy.

## Data Availability

The data presented in this study are available on request from the corresponding author. The data are not publicly available due to privacy restrictions.

## Appendix A

**Table A1:** Cell line information

Cell line	Origin	Mutation			Medium
		*KRAS*	*NRAS*	*BRAF*	
**Caco-2**	Colon adenocarcinoma	wt	wt	wt	HG-DMEM
**HCT116**	Colon carcinoma	G13D/wt	wt	wt	RPMI1640
**PANC-1**	Pancreatic ductal adenocarcinoma	G12D/wt	*NA*	wt	HG-DMEM
**DLD-1**	Colon adenocarcinoma	G13D/wt	*NA*	wt	HG-DMEM
**MIA PaCa-2**	Pancreatic ductal adenocarcinoma	G12C	*NA*	wt	HG-DMEM
**SW480**	Colon adenocarcinoma	G12V	*NA*	wt	HG-DMEM
**HT-29**	Colon adenocarcinoma	wt	wt	V600E/wt	HG-DMEM
**MOLT-4**	Adult T acute lymphoblastic leukemia	*NA*	G12C/wt	*NA*	HG-DMEM

Note: wt, *wild type*; NA, mutation not analyzed.

**Table A2:** dPCR protocol (Bio-Rad)

Cycling step	Temperature°C	Time	Ramp rate	Cycles
**Enzyme activation**	95	10 min	~2°C/sec	1
**Denaturation**	94	30 sec		40
**Annealing/extension**	55	1 min		
**Enzyme deactivation**	98	10 min		1
**Hold**	4	Infinite	~1°C/sec	1

**Table A3:** LoD of ddPCR for *KRAS* mutation detection in serial dilutions of genomic DNA

Concentration of gDNA (ng/mL)	Copies/µL
**100**	2.765 × 10^7^
**25**	6.912 × 10^6^
**6.25**	1.728 × 10^6^
**1.56**	4.313 × 10^5^
**0.39**	1.078 × 10^5^
**0.098**	2.710 × 10^4^
**0.024**	6.636 × 10^3^

Note: LoD, limit of detection; ddPCR, droplet digital PCR.

**Table A4:** Determination of mutations in a cohort of colorectal cancer (CRC) patients

ID	Conc. [ng/µL]	Mut KRAS predictive pathology	Mut NRAS predictive pathology	Mut BRAF predictive pathology	KRAS positive droplets (MUT)	KRAS negative droplets (MUT)	KRAS all droplets	MUT c	WT c	NRAS positive droplets (MUT)	NRAS negative droplets (MUT)	NRAS all droplets	MUT c	WT c	BRAF positive droplets (MUT)	BRAF negative droplets (MUT)	All droplets	MUT c	WT c
1	0.300	Undetected	Undetected	Undetected	5	37,398	37,403	0.16	26.30						0	49,940	49,940	0.00	23.70
2	0.572	Undetected	Undetected	Undetected	0	42,960	42,960	0.00	43.10						0	54,728	54,728	0.00	48.30
3	1.600	Undetected	Undetected	Undetected	3	47,707	47,710	0.07	96.60						0	52,699	52,699	0.00	145.30
4	1.640	Undetected	Undetected	Undetected	2	51,920	51,922	0.05	105.70						0	55,456	55,456	0.00	103.60
5	0.610	Undetected	Mut G13R (59%)	Undetected	2	56,945	56,947	0.04	37.00	3	29,760	29,763	0.12	1.90	0	53,309	53,309	0.00	41.40
6	1.050	Undetected	Undetected	Undetected	0	52,856	52,856	0.00	59.00						0	52,507	52,507	0.00	77.40
7	0.722	Undetected	Undetected	Undetected	0	53,621	53,621	0.00	45.90						0	50,154	50,154	0.00	59.10
8	1.300	Mut G12D (15%)	Undetected	Undetected	3	59,235	59,238	0.06	93.10										
9	0.472	Undetected	Undetected	Undetected	1	51,534	51,535	0.02	33.30						0	53,519	53,519	0.00	37.50
11	1.100	Undetected	Undetected	Undetected	2	55,396	55,398	0.04	76.60						0	51,001	51,001	0.00	87.10
12	0.421	Undetected	Undetected	Undetected	0	56,844	56,844	0.00	27.00						0	55,961	55,961	0.00	22.30
13	0.691	Mut G13D (8%)	Undetected	Undetected	2	54,732	54,734	0.04	57.40										
14	0.744	Undetected	Mut Q61R (30%)	Undetected	1	56,068	56,069	0.02	49.60	0	29,488	29,488	0.00	10.50	0	50,628	50,628	0.00	19.10
15	0.230	Mut G12D (21%)	Undetected	Undetected	35	43,584	43,619	0.94	19.30										
16	0.312	Mut G12D (23%)	Undetected	Not tested	3	50,754	50,757	0.07	36.20										
17	0.730	Undetected	Undetected	Undetected	1	50,963	50,964	0.02	92.60						0	37,898	37,898	0.00	14.90
18	2.960	Undetected	Undetected	Mut V600E (34%)	5	60,610	60,615	0.10	397.00						5	40,303	40,308	0.15	114.70
19	0.807	Mut G12V (34%)	Undetected	Undetected	5	58,744	58,749	0.10	83.90										
20	0.699	Mut G13D (30%)	Undetected	Undetected	5	58,021	58,026	0.10	111.50										
21	0.491	Mut G12D (30%)	Undetected	Undetected	7	55,852	55,859	0.15	41.50										
22	1.170	Undetected	Undetected	Mut V600E (19%)	3	39,073	39,076	0.09	79.50						2	39,267	39,269	0.06	54.40
23	1.290	Undetected	Mut G13V (29%)	Undetected	1	61,433	61,434	0.02	99.10	0	32,101	32,101	0.00	8.60					
24	1.160	Undetected	Undetected	Mut V600E (15%)	1	61,274	61,275	0.02	100.40						0	48,968	48,968	0.00	99.40
25	0.489	Mut G12F (38%)	Undetected	Undetected	0	41,113	41,113	0.00	41.70										
26	0.410	Undetected	Undetected	Undetected	0	61,555	61,555	0.00	26.60						0	46,302	46,302	0.00	5.10
27	0.587	Mut G12V (31%)	Undetected	Undetected	268	59,666	59,934	5.30	46.10										
28	0.541	Mut G13D (13%		Undetected	2	59,313	59,315	0.04	64.20										
29	0.150	Mut G12D (28%)	Undetected	Undetected	18	50,383	50,401	0.42	12.60										
30	0.451	Mut G13D (29%)	Undetected	Undetected	2	37,264	37,266	0.06	35.40										
32	0.954	Undetected	Undetected	Undetected	3	59,723	59,726	0.06	131.00						0	49,600	49,600	0.00	106.40
33	1.560	Mut G12S (57%)	Undetected	Undetected	3	41,444	41,447	0.09	58.80										
34	1.960	Mut G13D (40%)	Undetected	Undetected	1026	61,883	62,909	19.30	129.90										
35	0.339	Undetected	Undetected	Undetected	1	59,494	59,495	0.02	24.50						0	48,133	48,133	0.00	24.50
38	0.370	Mut G12D	Undetected	Undetected	1	38,422	38,423	0.03	23.30										
39	1.320	Mut G13D (43%)	Undetected	Undetected	644	34,640	35,284	21.70	68.50										
40	0.445	Undetected	Mut Q61R (78%)	Undetected	1	59,291	59,292	0.02	28.90	0	39,779	39,779	0.00	1.18	0	49,355	49,355	0.00	14.50
41	0.481	Undetected	Undetected	Undetected	0	57,531	57,531	0.00	29.90						0	53,315	53,315	0.00	36.00
42	0.184	Mut G13C (22%)	Undetected	Undetected	0	37,946	37,946	0.00	10.80										
43	1.420	Undetected	Undetected	Mut V600E (18%)	4	51,962	51,966	0.09	107.50						5	50,216	50,221	0.12	64.10
44	0.991	Undetected	Undetected	Mut V600E (29%)	0	54,668	54,668	0.00	68.40						0	50,380	50,380	0.00	72.90
45	0.383	Undetected	Undetected	Undetected	1	56,079	56,080	0.02	27.10						0	48,166	48,166	0.00	14.00
46	0.499	Undetected	Undetected	Mut V600E (22%)	2	55,811	55,813	0.04	38.00						329	53,315	53,644	1.62	7.20
47	0.371	Undetected	Undetected	Undetected	0	55,728	55,728	0.00	27.70	0	31,684	31,684	0.00	30.50	0	53,093	53,093	0.00	23.00
48	0.847	Mut G12D (22%)	Undetected	Undetected	255	55,465	55,720	5.40	53.70										
49	0.842	Undetected	Undetected	Mut V600E(28%)	3	59,603	59,606	0.06	64.70						4	49,995	49,999	0.09	67.00
50	0.242	Undetected	Undetected	Mut V600E	5	45,700	45,705	0.13	40.60										
52	0.773	Undetected	Undetected	Undetected	1	50,283	50,284	0.02	3.80	1	39,732	39,733	0.03	1.78					
53	0.230	Undetected	Undetected	Undetected	3	57,701	57,704	0.06	57.10	0	49,857	49,857	0.00	25.20	0	49,443	49,443	0.00	57.10
54	1.280	Undetected	Undetected	Undetected	3	58,938	58,941	0.06	15.50	0	47,118	47,118	0.00	0.37	0	51,605	51,605	0.00	13.40
55	1.270	Undetected	Undetected	Undetected	1	30,355	30,356	0.04	95.50	0	50,010	50,010	0.00	32.10	0	53,396	53,396	0.00	93.40
56	2.580	Undetected	Undetected	Undetected	1	52,813	52,814	0.02	0.00	0	47,853	47,853	0.00	33.80	0	53,025	53,025	0.00	139.00
57	33.900	Mut G12C	not tested	not tested	5	49,089	49,094	0.12	208.00	16	47,165	47,181	0.40	1030.00					
58	9.690	Undetected	Undetected	Undetected	31614	23,049	54,663	1016.00	1080.00						0	50,285	50,285	0.00	76.10
59	11.400	Mut G12V (25%)	Undetected	Undetected	10	59,773	59,783	0.20	217.70										
60	1.620	Mut G13C (30%)	Undetected	Undetected	7	39,260	39,267	0.21	828.00										
61	0.876	Undetected	Undetected	Mut V600E (20%)	5	59,190	59,195	0.10	113.30						0	52,941	52,941	0.00	50.70
62	0.440	Undetected	Undetected	Undetected	1	59,336	59,337	0.02	63.50						0	50,375	50,375	0.00	37.40
63	0.692	Mut G12V (38%)	Undetected	Undetected	1	39,078	39,079	0.03	32.60	3	50,761	50,764	0.07	24.40					
64	0.218	Undetected	Undetected	Undetected	0	55,845	55,845	0.00	0.34										
65	0.702	Mut G12D (30%)	Undetected	Undetected	0	56,589	56,589	0.00	11.90	1	49,483	49,484	0.02	25.30					
67	0.148	Mut A146T	Undetected	Undetected	0	40,862	40,862	0.00	0.63										
69	0.231	Undetected	Undetected	Undetected	4	55,287	55,291	0.09	13.30										
70	0.164	Mut G12A	Undetected	Undetected	0	54,719	54,719	0.00	0.15										
72	0.118	Mut A146T	Undetected	Undetected	5	54,853	54,858	0.11	9.90										
73	1.400	Mut Q61/E62	Undetected	Undetected	13	56,074	56,087	0.27	111.90										
74	1.610	Mut G12V	Undetected	Undetected	12	57,713	57,725	0.24	81.10										
75	0.105	Mut G12A	Undetected	Undetected	1	58,556	58,557	0.02	3.06										
76	0.252	Undetected	Undetected	Undetected	6	57,286	57,292	0.12	11.10										
77	0.161	Undetected	Undetected	Undetected	23	56,263	56,286	0.48	19.80										
78	0.114	Undetected	Undetected	Undetected	46	56,490	56,536	0.96	4.50										
79	0.253	Undetected	Undetected	Undetected	7	54,157	54,164	0.15	20.60										
80	0.625	Mut G12A	Undetected	Undetected	15	56,146	56,161	0.31	36.40										
81	1.530	Undetected	Undetected	Undetected	13	56,671	56,684	0.27	119.80										
82	0.630	Mut G12V	Undetected	Undetected	9	52,008	52,017	0.20	40.80										
83	0.646	Undetected	Undetected	Undetected	20	57,099	57,119	0.41	52.50										
84	0.553	Undetected	Undetected	Undetected	14	53,020	53,034	0.31	36.20										
85	0.145	Mut G12V	Undetected	Undetected	21	48,126	48,147	0.51	7.80										
86	0.673	Mut A146T	Undetected	Undetected	4	575,10	57,514	0.08	36.10										
87	2.640	Undetected	Undetected	Undetected	19	51,692	51,711	0.43	170.00										
88	0.468	Mut G12V	Undetected	Undetected	18	56,115	56,133	0.38	28.70										
89	0.837	Mut G12C	Undetected	Undetected	20	52,456	52,476	0.45	68.90										
90	0.415	Undetected	Undetected	Undetected	0	56,573	56,573	0.00	0.19										
91	2.500	Undetected	Undetected	Undetected	14	53,180	53,194	0.31	144.20										
94	0.310	Mut G12D	Undetected	Undetected	1	54,237	54,238	0.02	16.00										
95	0.113	Mut G13D	Undetected	Undetected	0	50,674	50,674	0.00	5.20										
97	0.672	Mut G12D	Undetected	Undetected	1	55,005	55,006	0.02	38.20										
98	0.935	Mut G12D	Undetected	Undetected	2	53,245	53,247	0.04	42.80										
99	0.730	Mut G12D	Undetected	Undetected	5	58,019	58,024	0.10	29.30										
100	0.369	Mut G12D	Undetected	Undetected	25	62,266	62,291	0.47	9.90										
101	7.410	Mut G12V	Undetected	Undetected	5080	56,380	61,460	101.50	173.10										
104	0.590	Undetected	Undetected	Undetected	0	59,701	59,701	0.00	31.40										
109	0.477	Mut G12S	Undetected	Undetected	153	56,638	56,791	3.20	25.50										
110	0.466	Undetected	Undetected	Undetected	0	57,731	57,731	0.00	25.10										
111	0.354	Mut G12C	Undetected	Undetected	0	51,801	51,801	0.00	15.50										
112	1.990	Mut G12V	Undetected	Undetected	3	56,261	56,264	0.06	145.20										
113	0.515	Undetected	Undetected	Undetected	0	59,179	59,179	0.00	22.80										
116	2.210	Mut G12S	Undetected	Undetected	11	55,384	55,395	0.23	175.20										
117	0.659	Undetected	Undetected	Undetected	0	58,275	58,275	0.00	31.10										
118	1.650	Undetected	Undetected	Undetected	12	59,918	59,930	0.28	104.30										
119	0.415	Undetected	Undetected	Undetected	0	62,630	62,630	0.00	4.20										
120	0.609	Undetected	Undetected	Undetected	13	60,954	60,967	0.25	37.40										
121	1.130	Undetected	Undetected	Undetected	7	54,513	54,520	0.15	74.10										
122	0.278	Undetected	Undetected	Undetected	0	59,264	59,264	0.00	12.80										
124	0.328	Undetected	Undetected	Undetected	8	59,765	59,773	0.12	11.20										
125	0.194	Undetected	Undetected	Undetected	10	60,070	60,080	0.21	14.40										
126	0.619	Mut G12C	Undetected	Undetected	4	58,360	58,364	0.08	31.60										
127	1.200	Undetected	Undetected	Undetected	0	60,856	60,856	0.00	72.20										
128	0.274	Mut G12C	Undetected	Undetected	1	60,653	60,654	0.02	9.90										
129	1.200	Mut G13D	Undetected	Undetected	0	59,768	59,768	0.00	65.70										
130	0.106	Mut G12D	Undetected	Undetected	5	57,561	57,566	0.10	6.40										

**Table A5:** Comparison of baseline patient characteristics according to the presence of KRAS mutation by NGS

Basic characteristics of patients and primary tumor (n = 110 patients)		Without KRAS mutation by NGS (n = 60)	Presence of KRAS mutation by NGS (n = 50)	*p*-value
**Sex**	Female	20 (33.3%)	19 (38.0%)	0.757
	Male	40 (66.7%)	31 (62.0%)	
**Age at surgery**	[years]	64.4 ± 10.6	64.6 ± 10.9	0.907
		65.8 (59.6; 73.1)	66.0 (56.7; 72.2)	
**Localization of the primary tumor**	Cecum (C18.0, C18.1)	9 (15.0%)	11 (22.0%)	0.134
	Colon (C18.2–C18.7, C18.9)	15 (25.0%)	13 (26.0%)	
	Lesions extending beyond the colon (C18.8)	5 (8.3%)	3 (6.0%)	
	Rectosigmoideal connection (C19)	17 (28.3%)	5 (10.0%)	
	Rectum (C20)	14 (23.3%)	18 (36.0%)	
**Extent of tumor (T)**	T1	0 (0.0%)	1 (2.0%)	0.587
	T2	6 (10.0%)	2 (4.0%)	
	T3	40 (66.7%)	32 (64.0%)	
	T4	13 (21.7%)	14 (28.0%)	
	Tx	1 (1.7%)	1 (2.0%)	
**Metastases in the lymph nodes (N)**	N0	24 (40.0%)	13 (26.0%)	0.270
	N1	19 (31.7%)	15 (30.0%)	
	N2	16 (26.7%)	21 (42.0%)	
	Nx	1 (1.7%)	1 (2.0%)	
**Distant metastases (M)**	M0	32 (53.3%)	25 (50.0%)	0.735
	M1	24 (40.0%)	23 (46.0%)	
	Mx	4 (6.7%)	2 (4.0%)	
**Lymphovascular space invasion (L)**	L0	33 (55.0%)	29 (58.0%)	0.659
	L1	21 (35.0%)	14 (28.0%)	
	Lx	6 (10.0%)	7 (14.0%)	
**Grade**	G1	15 (25.0%)	12 (24.0%)	0.986
	G2	33 (55.0%)	27 (54.0%)	
	G3	9 (15.0%)	9 (18.0%)	
	G4	1 (1.7%)	0 (0.0%)	
	Gx	2 (3.3%)	2 (4.0%)	
**Clinical stage**	Stage I	3 (5.0%)	0 (0.0%)	0.378
	Stage II	15 (25.0%)	9 (18.0%)	
	Stage III	18 (30.0%)	18 (36.0%)	
	Stage IV	24 (40.0%)	23 (46.0%)	
**Neoadjuvant therapy**	No	51 (85.0%)	42 (84.0%)	1.000
	Yes	9 (15.0%)	8 (16.0%)	

Note: Mean ± standard deviation and median (interquartile range) are given for continuous variables, and absolute (relative) frequencies are given for categorical variables. *p*-values for statistical significance of differences between groups were calculated using the Pearson chi-square test (for nominal variables), the *t*-test (for continuous variables), and the Mann–Whitney test (for ordinal variables). If the assumptions for the Pearson chi-square test or the *t*-test were not met, Fisher’s exact test or the Mann–Whitney test was used instead.

**Table A6:** Comparison of baseline patient characteristics according to the presence of any KRAS/BRAF/NRAS mutation by NGS

Basic characteristics of patients and primary tumor (n = 110 patients)		Without KRAS/BRAF /NRAS mutation by NGS (n = 47)	Presence of KRAS/BRAF /NRAS mutation by NGS (n = 63)	*p*-value*
**Sex**	Female	15 (31.9%)	24 (38.1%)	0.639
	Male	32 (68.1%)	39 (61.9%)	
**Age at surgery**	[years]	62.9 ± 11.0	65.7 ± 10.4	0.226
		64.3 (57.9; 71.0)	67.3 (58.7; 74.5)	
**Localization of the primary tumor**	Cecum (C18.0, C18.1)	4 (8.5%)	16 (25.4%)	**0.009**
	Colon (C18.2–C18.7, C18.9)	12 (25.5%)	16 (25.4%)	
	Lesions extending beyond the colon (C18.8)	4 (8.5%)	4 (6.3%)	
	Rectosigmoideal connection (C19)	16 (34.0%)	6 (9.5%)	
	Rectum (C20)	11 (23.4%)	21 (33.3%)	
**Extent of tumor (T)**	T1	0 (0.0%)	1 (1.6%)	0.226
	T2	6 (12.8%)	2 (3.2%)	
	T3	31 (66.0%)	41 (65.1%)	
	T4	9 (19.1%)	18 (28.6%)	
	Tx	1 (2.1%)	1 (1.6%)	
**Metastases in the lymph nodes (N)**	N0	22 (46.8%)	15 (23.8%)	**0.049**
	N1	13 (27.7%)	21 (33.3%)	
	N2	11 (23.4%)	26 (41.3%)	
	Nx	1 (2.1%)	1 (1.6%)	
**Distant metastases (M)**	M0	28 (59.6%)	29 (46.0%)	0.353
	M1	17 (36.2%)	30 (47.6%)	
	Mx	2 (4.3%)	4 (6.3%)	
**Lymphovascular space invasion (L)**	L0	29 (61.7%)	33 (52.4%)	0.620
	L1	13 (27.7%)	22 (34.9%)	
	Lx	5 (10.6%)	8 (12.7%)	
**Grade**	G1	15 (31.9%)	12 (19.0%)	0.233
	G2	25 (53.2%)	35 (55.6%)	
	G3	5 (10.6%)	13 (20.6%)	
	G4	1 (2.1%)	0 (0.0%)	
	Gx	1 (2.1%)	3 (4.8%)	
**Clinical stage**	Stage I	3 (6.4%)	0 (0.0%)	**0.017**
	Stage II	15 (31.9%)	9 (14.3%)	
	Stage III	12 (25.5%)	24 (38.1%)	
	Stage IV	17 (36.2%)	30 (47.6%)	
**Neoadjuvant therapy**	No	41 (87.2%)	52 (82.5%)	0.684
	Yes	6 (12.8%)	11 (17.5%)	

Note: Mean ± standard deviation and median (interquartile range) are given for continuous variables, and absolute (relative) frequencies are given for categorical variables. *p*-values for statistical significance of differences between groups were calculated using the Pearson chi-square test (for nominal variables), the *t*-test (for continuous variables), and the Mann–Whitney test (for ordinal variables). If the assumptions for the Pearson chi-square test or the *t*-test were not met, Fisher’s exact test or the Mann–Whitney test was used instead. * The *p*-values highlighted in bold are statistically significant, i.e., *p* < 0.05.

**Table A7:** valuation of the diagnostic accuracy of *KRAS* or any *KRAS/BRAF/NRAS* mutation detection by ddPCR—subgroup analysis

Diagnostic performance parameters	KRAS mutation	KRAS/BRAF/NRAS mutation
**Clinical stage I + II (n = 27)**		
**Area under the curve (AUC)**	0.611 (0.410; 0.813)	0.611 (0.410; 0.813)
**Sensitivity**	66.7 (33.3; 88.9)%	66.7 (33.3; 88.9)%
**Specificity**	55.6 (33.0; 76.0)%	55.6 (33.0; 76.0)%
**Positive predictive value**	42.9 (20.6; 68.4)%	42.9 (20.6; 68.4)%
**Negative predictive value**	76.9 (47.8; 92.4)%	76.9 (47.8; 92.4)%
**Clinical stage III (n = 36)**		
**Area under the curve (AUC)**	0.528 (0.360; 0.695)	0.583 (0.406; 0.760)
**Sensitivity**	55.6 (33.0; 76.0)%	58.3 (38.3; 75.9)%
**Specificity**	50.0 (28.4; 71.6)%	58.3 (30.8; 81.5)%
**Positive predictive value**	52.6 (31.1; 73.2)%	73.7 (50.2; 88.6)%
**Negative predictive value**	52.9 (30.3; 74.5)%	41.2 (21.0; 64.8)%
**Clinical stage IV (n = 47)**		
**Area under the curve (AUC)**	0.643 (0.520; 0.766)	0.623 (0.484; 0.761)
**Sensitivity**	87.0 (66.5; 95.7)%	83.3 (65.7; 92.9)%
**Specificity**	41.7 (24.1; 61.7)%	41.2 (21.0; 64.8)%
**Positive predictive value**	58.8 (41.9; 73.9)%	71.4 (54.6; 83.9)%
**Negative predictive value**	76.9 (47.8; 92.4)%	58.3 (30.8; 81.5)%
**Without neoadjuvant therapy (n = 93)**		
**Area under the curve (AUC)**	0.658 (0.564; 0.751)	0.665 (0.569; 0.761)
**Sensitivity**	78.6 (63.7; 88.5)%	76.9 (63.6; 86.4)%
**Specificity**	52.9 (39.4; 66.1)%	56.1 (40.8; 70.3)%
**Positive predictive value**	57.9 (44.8; 69.9)%	69.0 (56.0; 79.5)%
**Negative predictive value**	75.0 (58.5; 86.4)%	65.7 (48.8; 79.4)%

Note: For all values 95% CI is given.

**Table A8:** Comparison of baseline patient characteristics according to the presence of KRAS mutation by NGS

Basic characteristics of patients and primary tumor (n = 110 patients)		Without KRAS mutation by NGS (n = 60)	Presence of KRAS mutation by NGS (n = 50)	*p*-value
**Sex**	Female	20 (33.3%)	19 (38.0%)	0.757
	Male	40 (66.7%)	31 (62.0%)	
**Age at surgery**	[years]	64.4 ± 10.6	64.6 ± 10.9	0.907
		65.8 (59.6; 73.1)	66.0 (56.7; 72.2)	
**Localization of the primary tumor**	Cecum (C18.0, C18.1)	9 (15.0%)	11 (22.0%)	0.134
	Colon (C18.2–C18.7, C18.9)	15 (25.0%)	13 (26.0%)	
	Lesions extending beyond the colon (C18.8)	5 (8.3%)	3 (6.0%)	
	Rectosigmoideal connection (C19)	17 (28.3%)	5 (10.0%)	
	Rectum (C20)	14 (23.3%)	18 (36.0%)	
**Extent of tumor (T)**	T1	0 (0.0%)	1 (2.0%)	0.587
	T2	6 (10.0%)	2 (4.0%)	
	T3	40 (66.7%)	32 (64.0%)	
	T4	13 (21.7%)	14 (28.0%)	
	Tx	1 (1.7%)	1 (2.0%)	
**Metastases in the lymph nodes (N)**	N0	24 (40.0%)	13 (26.0%)	0.270
	N1	19 (31.7%)	15 (30.0%)	
	N2	16 (26.7%)	21 (42.0%)	
	Nx	1 (1.7%)	1 (2.0%)	
**Distant metastases (M)**	M0	32 (53.3%)	25 (50.0%)	0.735
	M1	24 (40.0%)	23 (46.0%)	
	Mx	4 (6.7%)	2 (4.0%)	
**Lymphovascular space invasion (L)**	L0	33 (55.0%)	29 (58.0%)	0.659
	L1	21 (35.0%)	14 (28.0%)	
	Lx	6 (10.0%)	7 (14.0%)	
**Grade**	G1	15 (25.0%)	12 (24.0%)	0.986
	G2	33 (55.0%)	27 (54.0%)	
	G3	9 (15.0%)	9 (18.0%)	
	G4	1 (1.7%)	0 (0.0%)	
	Gx	2 (3.3%)	2 (4.0%)	
**Clinical stage**	Stage I	3 (5.0%)	0 (0.0%)	0.378
	Stage II	15 (25.0%)	9 (18.0%)	
	Stage III	18 (30.0%)	18 (36.0%)	
	Stage IV	24 (40.0%)	23 (46.0%)	
**Neoadjuvant therapy**	No	51 (85.0%)	42 (84.0%)	1.000
	Yes	9 (15.0%)	8 (16.0%)	

Note: Mean ± standard deviation and median (interquartile range) are given for continuous variables, and absolute (relative) frequencies are given for categorical variables. *p*-values for statistical significance of differences between groups were calculated using the Pearson chi-square test (for nominal variables), the *t*-test (for continuous variables), and the Mann–Whitney test (for ordinal variables). If the assumptions for the Pearson chi-square test or the *t*-test were not met, Fisher’s exact test or the Mann–Whitney test was used instead.

**Table A9:** Comparison of baseline patient characteristics according to the presence of any KRAS/BRAF/NRAS mutation by NGS

Basic characteristics of patients and primary tumor (n = 110 patients)		Without KRAS/BRAF /NRAS mutation by NGS (n = 47)	Presence of KRAS/BRAF /NRAS mutation by NGS (n = 63)	*p*-value*
**Sex**	Women	15 (31.9%)	24 (38.1%)	0.639
	Men	32 (68.1%)	39 (61.9%)	
**Age at surgery**	[years]	62.9 ± 11.0	65.7 ± 10.4	0.226
		64.3 (57.9; 71.0)	67.3 (58.7; 74.5)	
**Localization of the primary tumor**	Cecum (C18.0, C18.1)	4 (8.5%)	16 (25.4%)	**0.009**
	Colon (C18.2–C18.7, C18.9)	12 (25.5%)	16 (25.4%)	
	Lesions extending beyond the colon (C18.8)	4 (8.5%)	4 (6.3%)	
	Rectosigmoideal connection (C19)	16 (34.0%)	6 (9.5%)	
	Rectum (C20)	11 (23.4%)	21 (33.3%)	
**Extent of tumor (T)**	T1	0 (0.0%)	1 (1.6%)	0.226
	T2	6 (12.8%)	2 (3.2%)	
	T3	31 (66.0%)	41 (65.1%)	
	T4	9 (19.1%)	18 (28.6%)	
	Tx	1 (2.1%)	1 (1.6%)	
**Metastases in the lymph nodes (N)**	N0	22 (46.8%)	15 (23.8%)	**0.049**
	N1	13 (27.7%)	21 (33.3%)	
	N2	11 (23.4%)	26 (41.3%)	
	Nx	1 (2.1%)	1 (1.6%)	
**Distant metastases (M)**	M0	28 (59.6%)	29 (46.0%)	0.353
	M1	17 (36.2%)	30 (47.6%)	
	Mx	2 (4.3%)	4 (6.3%)	
**Lymphovascular space invasion (L)**	L0	29 (61.7%)	33 (52.4%)	0.620
	L1	13 (27.7%)	22 (34.9%)	
	Lx	5 (10.6%)	8 (12.7%)	
**Grade**	G1	15 (31.9%)	12 (19.0%)	0.233
	G2	25 (53.2%)	35 (55.6%)	
	G3	5 (10.6%)	13 (20.6%)	
	G4	1 (2.1%)	0 (0.0%)	
	Gx	1 (2.1%)	3 (4.8%)	
**Clinical stage**	Stage I	3 (6.4%)	0 (0.0%)	**0.017**
	Stage II	15 (31.9%)	9 (14.3%)	
	Stage III	12 (25.5%)	24 (38.1%)	
	Stage IV	17 (36.2%)	30 (47.6%)	
**Neoadjuvant therapy**	No	41 (87.2%)	52 (82.5%)	0.684
	Yes	6 (12.8%)	11 (17.5%)	

Note: Mean ± standard deviation and median (interquartile range) are given for continuous variables, and absolute (relative) frequencies are given for categorical variables. *p*-values for statistical significance of differences between groups were calculated using the Pearson chi-square test (for nominal variables), the *t*-test (for continuous variables), and the Mann–Whitney test (for ordinal variables). If the assumptions for the Pearson chi-square test or the *t*-test were not met, Fisher’s exact test or the Mann–Whitney test was used instead. * The *p*-values highlighted in bold are statistically significant, i.e., *p* < 0.05.
